# ‘Plugging the gap’: development of a plain language glossary for statistical methodology research

**DOI:** 10.1186/s40900-025-00782-4

**Published:** 2025-10-14

**Authors:** Sarah Booth, Molly Wells, Clareece Nevill, Lucy Teece, Barbara Czyznikowska, Gurpreet Grewal-Santini, Mary Mancini, Farheen Yameen, Suzanne C Freeman

**Affiliations:** 1 Biostatistics Research Group, Department of Population Health Sciences, University of Leicester, Leicester, UK; 2 Division of Informatics, Imaging and Data Science, Faculty of Biology, Medicine and Health, University of Manchester, Manchester, UK; 3 Manchester Academic Health Science Centre, Manchester, UK; 4 AstraZeneca, Macclesfield, UK; 5 Centre for Ethnic Health Research, University of Leicester, Leicester, UK; 6 Public Contributor, PPI-SMART (Patient and Public Involvement for Statistical Methodology and Research Techniques), University of Leicester, Leicester, UK

**Keywords:** Patient and public involvement and engagement (PPIE), Statistical methodology research, Communication

## Abstract

**Background:**

Plain language glossaries of research-related terms are a useful resource for Patient and Public Involvement and Engagement (PPIE) activities. They can provide public contributors with a deeper understanding of aspects of the project such as the study design, the methods being used for data analysis, and the interpretation of results. However, whilst plain language glossaries of research-related terms exist, they do not always include definitions for concepts that are commonly used in statistical methodology research. The aims of this work were to (1 develop a plain language glossary of the statistical methodology research terms that were missing from the existing glossaries and 2) outline the process used to develop the glossary to aid researchers in producing a glossary relevant to their own research projects.

**Methods:**

By conducting online searches and consulting members of the Biostatistics Research Group at the University of Leicester, we conducted a scoping exercise in August 2023 to identify glossaries aimed at members of the public that included definitions of statistical terms. We then reviewed the glossaries to develop a list of terms that are commonly used in the statistical methodology research conducted by the Biostatistics Research Group at the University of Leicester, which had not already been defined. Initial definitions of these terms were generated using ChatGPT (GPT-3.5). These were then refined and discussed with public contributors. Three cycles of PPIE feedback were used to further develop and update the definitions for use in the glossary.

**Results:**

We reviewed gaps in five existing glossaries and identified a list of 64 statistical terms to develop definitions for. These covered a range of concepts including different types of statistical models, Bayesian analysis, meta-analysis, and prognostic modelling. The feedback we received from public contributors focused on the level of language used, shortening the length of the definitions, and including examples to give context to the definitions.

**Conclusions:**

We developed a plain language glossary of terms that are commonly used in statistical methodology research as a resource for public contributors taking part in PPIE. The glossary has been made available on the NIHR Leicester Biomedical Research Centre website (https://leicesterbrc.nihr.ac.uk/ppismart/ppismart-definitions/).

**Supplementary Information:**

The online version contains supplementary material available at 10.1186/s40900-025-00782-4.

## Abstract (plain language summary)

Statistical methodology research focuses on finding the best tools to analyse data and making sure they work well. It is important for members of the public to be involved in statistical methodology research. To help and encourage people to get involved, researchers at the University of Leicester have been making resources to help explain what statistical methodology research is.

Glossaries of research terms are a simple way for members of the public to understand what is going on and join in. We found five glossaries that included some words we often use but there were many words missing. The aim of this project was to create a new glossary that included the words that are often used by researchers working in statistical methodology research but were not already included in existing glossaries.

This paper explains the process of developing the glossary. First definitions were created using ChatGPT (GPT-3.5). Feedback from members of the public was then used to update the definitions. We repeated the feedback and updating cycle three times. This made sure that the definitions (i) were using the right level of language, (ii) were made up of short sentences, and (iii) included examples.

The final glossary includes simple definitions for 64 terms that are commonly used by statistical methodology researchers. The glossary is freely available on the NIHR website for anyone to use (https://leicesterbrc.nihr.ac.uk/ppismart/ppismart-definitions/). We hope that other researchers find the glossary a useful resource to help keep members of the public engaged with their research projects.

**Supplementary Information:**

The online version contains supplementary material available at 10.1186/s40900-025-00782-4.

## Background

The incorporation and requirement of Patient and Public Involvement and Engagement (PPIE) in health research (including applications and proposals) [1] has increased in recent years, but this has typically been in applied studies (such as clinical trials), rather than methods-based research (such as simulation studies) [2]. Statistical methodology research, in which the statistical tools used in applied studies are refined and improved [3] is essential for improving health outcomes in both the local and global community. It can improve patient care and the health of the public, which also benefits society and the economy. Statistical methodology research conducted by the Biostatistics Research Group at the University of Leicester is used within health technology assessments and clinical guidelines which can change healthcare policy, the way care is provided and the interventions received. It is therefore essential that PPIE is also incorporated within statistical methodology research to ensure that the research conducted is relevant and applicable to as many people as possible. In the UK, organisations such as the National Institute for Health and Care Research (NIHR) and the Medical Research Council (MRC) have now made PPIE a requirement for all researchers in receipt of their funding (recommendation 8 of MRC Public Involvement Review [4]).

A survey assessing the current practices and attitudes of researchers towards PPIE in statistical methodology research identified that whilst most researchers thought that PPIE was beneficial to their research projects, some felt that statistical methodology research was too technical for the public to understand, and most felt they would feel more confident if they had access to resources specifically designed for statistical methodology research [3]. The PPI-SMART (PPI for Statistical Methodology and Research Techniques) group, formed in 2022 and made up of researchers in the Biostatistics Research Group and the Centre for Ethnic Health Research at the University of Leicester, have been working to improve the incorporation of PPIE in statistical methodology research and increase the resources available to facilitate successful PPIE. In previous work, the group has collaborated with Nifty Fox Creative to develop an animation (https://www.learningforinvolvement.org.uk/content/resource/what-is-statistical-methodology-research-and-why-is-ppie-input-important/) that defines and explains the concept of statistical methodology research and how PPIE can aid this research [5].

A Google search for ‘plain English glossary of statistical methodology research terms’ returns numerous results suggesting that resources may already exist for researchers to help the public to understand the key concepts within a statistical methodology research project [6–12]. However, delving into these glossaries further, reveals that many are actually aimed at people with some understanding of statistics [11] or omit some of the common terms encountered by members of PPI-SMART [10, 12]. Therefore, for researchers, the main barrier to conducting meaningful PPIE in statistical methodology research remains the struggle to communicate statistics in an accessible way, and, for the public, a lack of exposure to the concepts of statistical methodology and complex statistical terms. Furthermore, members of the public will likely have different backgrounds which will shape their understanding of research and so clear unified definitions are integral to their involvement [13].

### Aims

The aim of this work was to develop a plain language glossary of statistical methodology research terms, missing from existing glossaries, that was accessible to members of the public and could help facilitate meaningful PPIE in future statistical methodology research projects. Whilst we have developed definitions for many commonly used terms, statistical methodology is a broad field and researchers may find that there are some terms they use which are not included. Therefore, a secondary aim was to outline the process for developing this glossary to aid researchers in producing a glossary relevant to their own statistical methodology research projects. This process could equally be applied for non-statistical methodology projects that require a knowledge-specific glossary.

The aim of the PPIE in this study was to gain feedback on the definitions to ensure that the glossary produced would be suitable for PPIE representatives taking part in statistical methodology projects and would be useful for aiding their understanding of this research area.

## Methods and Results

### Setting

This project ran from July 2023 to August 2024 and was conducted by a working group of researchers in the PPI-SMART group based at the University of Leicester. The project involved two PPIE groups (further details below). The GRIPP2 (Guidance for Reporting Involvement of Patients and the Public) checklist [14] was followed and completed (see Supplementary Material 1).

### Development of the glossary

**Fig. 1:**
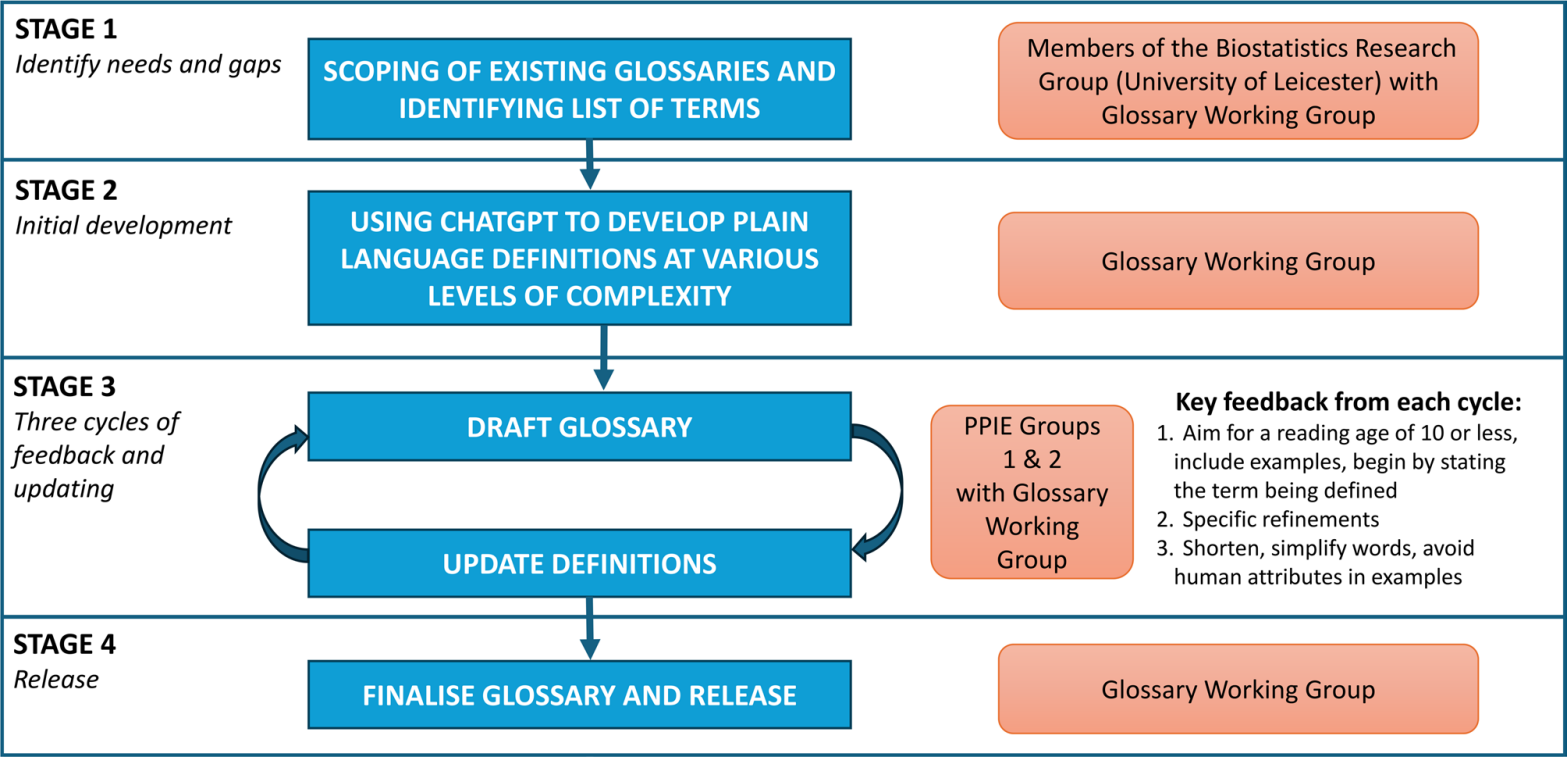
Flow diagram for the process of developing the plain language glossary for statistical methodology research

The stages taken in the development of the plain language glossary for statistical methodology research are summarised in Fig. 1 and described in detail below.

#### Stage 1: Identify needs and gaps

We first conducted a scoping exercise to identify any existing glossaries that included terms relevant to statistical methodology research and were explained in a manner that could be considered accessible to members of the public. Members of the Biostatistics Research Group at the University of Leicester (45–50 members including academic staff, research staff and PhD students) were asked to identify any glossaries they had previously used within their methodological research projects which included definitions of statistical terms accessible to PPIE members. In addition, Google searches were performed using search terms such as “plain English glossary of statistical terms” and “simple statistics definitions” in August 2023. Due to resource restraints, only glossaries published in English were considered. Assessment of whether a glossary was accessible to members of the public was made by PPI-SMART team members with experience of conducting PPIE.

In total, five existing glossaries were identified that included terms commonly used within statistical methodology research and were considered accessible to non-statisticians/researchers (although to varying levels):

- UK National Institute for Health and Care Excellence (NICE) Glossary (https://www.nice.org.uk/Glossary) [6].
- UK National Institute for Health and Care Research (NIHR) Glossary (https://www.nihr.ac.uk/glossary) [7].
- British Medical Journal (BMJ) Best Practice glossary of evidence-based medicine terms (https://bestpractice.bmj.com/info/us/evidence/ebm-tools/a-glossary-of-ebm-terms/) [8].
- NIHR Imperial Clinical Research Facility Glossary of Research Terms (https://www.imperial.ac.uk/media/imperial-college/medicine/imperial-crf/CRF-Glossary-of-research-terms.pdf) [9].
- Health Technology Assessment (HTA) glossary (https://htaglossary.net/List-all-terms) [10].

To identify whether there was a need for a new glossary, we asked all members of the Biostatistics Research Group to identify any terms they felt were relevant to statistical methodology research which were missing from the existing glossaries. As a result, we ended up with 64 terms to define within our glossary which are listed in Supplementary Material 2.

#### Stage 2: Initial development

In England, most adults have a reading age between 11 and 14 years old [15]. Therefore, we aimed to use a reading age of less than 10 years old to ensure that the language used was widely accessible. This is consistent with recommendations of using a reading age of 9–11 years old when communicating research and health information to the public [16, 17]. ChatGPT (GPT-3.5) [18] was used to generate an initial definition for each term in the glossary using the phrase “Create a definition for the statistical concept “X” aimed at a reading age of less than 10 years old using a maximum of 3 sentences”. If needed, the definitions were regenerated and phrases from each iteration were combined to produce one overall definition.

After reviewing this initial draft, the working group felt that whilst using an example to explain the statistical concept was useful, specifying the definition to be of a reading age less than 10 years old sometimes led to the examples being aimed at children. For example, an estimand was described as being like a “treasure map for statisticians…that guides them on their data adventure” and external validation was “like how we might ask a friend to double-check our math homework”. After removing the reading age condition, or instead specifying that it should be written in “plain English”, the examples given were considered to be more appropriate for the target audience and these were used in later iterations of the glossary.

In other cases, such as when generating definitions for “hazard” or “censoring” in survival analysis, the reading age of less than 10 years old had to be relaxed otherwise the statistical meaning of the word was not adequately captured. In these cases, the reading age was either raised incrementally until the statistical meaning was explained correctly, or removed completely.

In order to determine the level of language that should be used in our glossary, we prepared definitions for six terms of varying levels of complexity to discuss with PPIE members. In addition to the definition aimed at a reading age of less than 10 years old, we generated two further definitions for each term using ChatGPT, one written in “plain English” and another which was “suitable for a statistician” (see Table 1).

**Table 1:** Definitions generated at varying levels of complexity for six terms discussed during the first meeting with PPIE group 1

Term	Reading age of less than 10 years old	Plain English	Aimed at a statistician
Continuous outcome	A continuous outcome is a way of saying that something can be any number, like how tall you are or how much you weigh.	A “continuous outcome” in statistics refers to a type of data that can take any value within a given range. Examples of continuous outcomes include measurements like height, weight and temperature.	A continuous outcome, in statistics, refers to a type of variable that can take on an infinite number of values within a certain range. It is characterized by being measurable and having no distinct, separate categories. Examples of continuous outcomes include measurements like height, weight, temperature, and income.
Regression	Regression is a tool that helps us understand how things work together. Imagine you have lots of points on a graph, like dots on paper. Regression helps us draw a line through those points to see how they relate to each other.	Regression is a statistical method that helps us understand and predict the relationship between two or more variables. It’s like drawing a line through data points to find a pattern or trend, making it easier to make informed predictions or uncover hidden connections. Regression is widely used in fields like economics, science, medicine, and social sciences to analyse and make sense of data.	Regression is a statistical method that aims to model the relationship between a dependent variable and one or more independent variables. It provides a way to quantify and understand the association between these variables, allowing for predictions and inferences. Regression analysis seeks to find the best-fitting linear or nonlinear equation that describes this relationship, making it a fundamental tool for statistical modelling and hypothesis testing.
Hazard	In statistics, a “hazard” refers to the chance or risk of something happening, like an event or an outcome. It helps us understand how likely it is for a specific event to occur over time. Hazards are often used in survival analysis to study things like how long it takes for people to get sick.	In statistics, a “hazard” refers to the likelihood of an event, like an accident or failure, happening at a specific point in time. It helps us understand how the risk of that event changes over time, making it a valuable tool for predicting and managing potential threats or failures. Hazards are often used in survival analysis to assess how long it might take for an event to occur.	In statistics, a “hazard” is a fundamental concept in survival analysis. It represents the instantaneous rate of occurrence of an event, such as failure or death, at a specific point in time given that it has not occurred before that time. Hazards are crucial for modelling time-to-event data and understanding the dynamics of event occurrence over time.
Likelihood	Likelihood helps us think about the chances of different things occurring. When something has a high likelihood, it means it’s more likely to happen, and when it has a low likelihood, it’s not very likely to happen.	Likelihood in statistics tells us how probable or possible something is. It helps us understand the chances of different outcomes when we’re trying to figure out what’s happening or make predictions. A higher likelihood means something is more likely to occur, while a lower likelihood means it’s less likely.	In statistics, likelihood is a fundamental concept used to express how well a particular model or set of parameters explains observed data. It quantifies the probability of obtaining the observed data given a specific model or parameter values. Statisticians use likelihood to estimate and compare different models or parameters to find the best fit for the data.
MCMC	Markov Chain Monte Carlo, or MCMC for short, helps scientists figure out hard maths problems and make predictions about things. It’s like taking little steps to explore data and find the answers they need. Imagine you’re in a maze, and you can only move one step at a time, but you choose where to go randomly. MCMC helps scientists and researchers solve tricky puzzles by taking these random steps to explore a big maze and find answers they’re looking for.	Markov Chain Monte Carlo (MCMC) is a method in statistics and mathematics that helps us solve complex problems and make predictions. It works by taking lots of random steps to explore and understand big sets of data. People use it to find answers and make decisions in a wide range of fields, from science to finance.	Markov Chain Monte Carlo (MCMC) is a powerful numerical technique used in statistics and computational science to approximate complex probability distributions. It relies on iterative sampling, often involving random walks through high-dimensional spaces, to estimate hard-to-calculate statistics or make Bayesian inferences. MCMC is valuable for solving problems in fields such as Bayesian statistics, machine learning, and simulation.
Flexible parametric model	A flexible parametric model is a tool that helps us understand and predict things using numbers. It’s like drawing lines that can bend and twist to fit the data just right. It’s helpful when we want to learn more about different things and how they’re connected.	A flexible parametric model is a statistical tool that allows researchers to capture and describe a wide range of complex patterns in data. Unlike rigid models with fixed shapes, flexible parametric models can adapt and fit themselves to various data patterns, making them versatile for analysing diverse datasets. They are particularly useful for uncovering hidden relationships and understanding the underlying structure in data.	A flexible parametric model is a statistical framework that employs adjustable and versatile functions to describe data patterns. It provides statisticians with the ability to model complex relationships and distributions by using parameters that can adapt and fit the data more precisely. This approach is especially valuable when dealing with intricate datasets that cannot be adequately characterized by simpler, fixed-form models.

#### Stage 3: Cycles of PPIE feedback and updates

To identify the level of technicality at which to pitch our glossary, we first met with ‘PPIE Group 1’ online. PPIE Group 1 was a diverse group of five members of the public from different geographical areas. The group had a mixture of protected characteristics (e.g. age, race, sex) and included members for whom English was not their first language. This group had previously assisted with PPIE conducted for other PPI-SMART projects and therefore had some exposure to statistics and statistical methodology research.

We used the definitions we developed for six terms written at various levels of complexity as a tool to guide our discussion (see Table 1). We discussed differences in the definitions generated based on the level of language and reading proficiency to determine their preference for what level of language should be used.

Overall, the feedback was positive, and the group validated the need for a plain language glossary to be used to facilitate PPIE in statistical research. The key outcome was that the definitions should be written for a reading age of less than 10 years old, rather than written in plain English. The majority of PPIE members stated that they struggled to understand the definitions when they were written in plain English and felt that they could obtain a more thorough understanding of the statistical terms when they were pitched at a more basic level. In addition, some PPIE members identified that writing the definitions for a reading age of less than 10 would make the definitions more easily understood by people for whom English is not their first language. Furthermore, some PPIE members felt that whilst the plain English definitions were good for documentation, the reading age of less than 10 was better for understanding. PPIE members identified that the start of the definition needed to directly state what the respective term was, which some definitions initially did not. To aid understanding of definitions, the PPIE members identified that examples are needed to help illustrate the terms and solidify understanding. Finally, it was raised that the inclusion of some form of infographic or visual aid as part of the glossary would further elevate the definitions and ensure that the glossary was also accessible for those that are neurodiverse or have learning difficulties.

The working group then updated the initial version of the glossary based on the feedback from PPIE Group 1 (summarised above) giving a first revision of the glossary. Key changes included ensuring that definitions had examples and began by directly stating the term being defined. Each member of the working group updated different definitions to the ones they initially generated, and all members were given the opportunity to comment on and refine each other’s definitions.

PPIE Group 1 were then asked if they would review the updated glossary and all five members responded positively. Each member was sent a selection of definitions from the updated glossary by email, such that no member had the exact same list, yet every definition was reviewed by two members, and asked to email back with their feedback.

Three members gave feedback for specific definitions which led to further revisions of the glossary. Working group members were assigned to each PPIE member’s comments and worked in groups of twos or threes to consider the suggestions for each definition. The feedback was incorporated as far as possible whilst ensuring the definitions remained correct, leading to a second revision of the glossary.

We later met with a second PPIE group, ‘PPIE Group 2’, online to gain further feedback on the glossary. This was a diverse group of five members with a mixture of protected characteristics (e.g. age, race, sex) and included members for whom English is not their first language. Whilst all members of the group had prior experience of PPIE in applied research contexts (e.g. reviewing documentation, contributing to the study design, shaping dissemination pathways), none had previously participated in statistical methodology research. This group was recruited through the Centre for Ethnic Health Research (CEHR). Each member was asked to review the glossary in advance and to provide feedback during the meeting.

There were three key areas of discussion and feedback from this meeting:

1. Most members felt that the definitions were too long and should be shortened.
2. Some members felt that terms such as “average”, “accurate” and “prediction” were too technical.
3. All members agreed that whilst using examples helped them to understand the definitions, they preferred examples which did not use human attributes such as height or weight since these could be stigmatising. Additionally, they felt that people may find it difficult to relate to an example if it is based on a characteristic or event that they have not experienced. Instead, members suggested using examples such as plant heights which they felt would be relatable to everyone.

The glossary was then updated based on the group’s feedback to shorten and simplify the definitions and ensure that they included suitable examples. As far as possible, terms which were highlighted as being too technical were changed, however in certain definitions it was felt that changing “prediction” to something like “guess” would lose the concept of the definition. After the updates, the glossary was then reviewed and proofread by all members of the working group. Table 2 provides an example of how the definition of “regression” developed over time by implementing feedback from PPIE Groups 1 and 2.

**Table 2:** The initial and updated definitions of the term “regression” following feedback from PPIE Groups 1 and 2

Initial definition (reading age of less than 10 years old)	Updated definition following feedback from PPIE Group 1	Updated definition following feedback from PPIE Group 2
Regression is a tool that helps us understand how things work together. Imagine you have lots of points on a graph, like dots on paper. Regression helps us draw a line through those points to see how they relate to each other.	Regression is a tool that helps us understand how numbers and other information work together. Imagine you have a lot of points on a graph, like dots on paper, and each point represents the height and age of an individual person. Regression helps us draw a line through those points to see how they relate to each other. It’s like connecting the dots to figure out a pattern.	Regression is a tool that helps us find patterns in data. Imagine you have a lot of points on a graph, showing the height and number of leaves on a plant. Regression helps us draw a line through those points to see how they relate to each other.

#### Stage 4: Release

The glossary has been made publicly available on the NIHR Leicester Biomedical Research Centre website (https://leicesterbrc.nihr.ac.uk/ppismart/ppismart-definitions/) and can be downloaded as an editable Word document to allow researchers to just select the terms that are relevant to their research.

As recommended by the PPIE representatives, ongoing work to the glossary includes designing complementary visualisations for terms to further help with the explanation of certain statistical concepts.

### Recommendations for the development of future glossaries

Based on our experience developing the glossary, we have developed some recommendations for any researchers who are considering writing their own definitions of statistical terms to supplement existing glossaries, Box 1.

**Table:** 

**Box. 1** Recommendations for developing a glossary of statistical terms	
1. Aim for a reading age of less than 10 years old (or equivalent reading level)	
2. Include easy-to-relate-to examples avoiding the use of human attributes to help explain statistical concepts	
3. Pilot a few definitions with a PPIE group to check their suitability before drafting the full glossary	

## Discussion and Conclusion

We developed a plain language glossary of statistical methodology research terms to facilitate meaningful PPIE in future statistical methodology research projects. The glossary is freely available at https://leicesterbrc.nihr.ac.uk/ppismart/ppismart-definitions/.

Our glossary is not an exhaustive list of statistical methodology research terms. It aims to ‘plug-the-gaps’ of existing glossaries by defining terms that are commonly used within statistical methodology research that were not included in existing glossaries. We recommend that when researchers engage with PPIE members about a new project they provide PPIE members with some guidance on using the glossary. For example, the glossary covers many terms from the two main research areas of the Biostatistics Research Group at the University of Leicester, namely: survival analysis and evidence synthesis. Therefore, researchers may wish to identify specific terms of relevance to their projects and also guide PPIE members to other glossaries with relevant terms [6–10, 12]. Furthermore, our glossary should not be considered as the only resource for PPIE members involved in statistical methodology research. It should be considered as one tool amongst many for building PPIE capacity within statistical methodology research and as an aid to help facilitate meaningful PPIE in this field. By utilising technology, such as the *glossary* package in R [19] future resources could be developed such that definitions are embedded within (online) PPIE research documents so that PPIE members can follow along more easily.

We found that ChatGPT was a useful tool for generating an initial definition although restrictions on the reading age may need to be relaxed for some terms in order to adequately capture their statistical meaning. In addition, using a reading age of less than 10 years old sometimes led to the examples used in the definition being aimed at children. During the peer review process, a reviewer raised whether specifying the level of language rather than a reading age may help to prevent against this. Based on some initial results using the prompt “Create a definition for the statistical concept “X” in Entry Level 3 language using a maximum of 3 sentences” (with GPT-4), this method seemed to work well. We would therefore recommend any researchers developing their own glossary to consider and investigate this further.

The PPIE conducted in this project was highly impactful and allowed us to make substantial improvements to the glossary both in terms of the content and language used. We found that working with two PPIE groups that had varying levels of previous involvement in statistical methodology research projects was useful for understanding how the glossary may be received by those who are new to the area and those who already have some familiarity with this type of research. However, whilst not all PPIE members had prior experience of PPIE for statistical methodology research, all members had previously participated in PPIE activities for applied research. We recognise this as a limitation and that, if possible, it would have been beneficial to have also included members of the public who were completely new to PPIE.

Whilst we made substantial updates to the definitions based on PPIE feedback, there were some limitations to implementing all of the feedback. For example, we were unable to avoid using terms such as “prediction” for some definitions without losing their statistical meaning. In addition, some PPIE members felt that some of the definitions would benefit from an accompanying visualisation but at the time of the glossary development this was not possible due to resource constraints.

However, subsequently a funding source was identified and work is ongoing to develop visualisations for the glossary. As part of the PPIE for this, we have received additional feedback on some of the definitions which we have used to further refine the glossary. We will publish an updated version of the glossary shortly along with the visualisations.

Statistical methodology research plays an important role in improving health outcomes and guiding healthcare policies. It is therefore essential that PPIE is incorporated into this type of research; glossaries such as the one developed here are integral to facilitating meaningful PPIE in this field.

## Supplementary Information

Below is the link to the electronic supplementary material.

Supplementary Material 1

Supplementary Material 2

## Data Availability

Our glossary is available online at: https://leicesterbrc.nihr.ac.uk/ppismart/ppismart-definitions/.

## Abbreviations

PPIE: Patient and Public Involvement and Engagement
PPI-SMART: Patient and Public Involvement for Statistical Methodology and Research Techniques
NIHR: National Institute for Health and Care Research
MRC: Medical Research Council
