# Supplementary material for: ‘Plugging the gap’: development of a plain language glossary for statistical methodology research
Source: Res Involv Engagem. 2025 Oct 14;11:118. doi: 10.1186/s40900-025-00782-4 (PMC12523162; doi:10.1186/s40900-025-00782-4)
Supplement: Supplementary file 1 — Supplementary Material 1 [file 40900_2025_782_MOESM1_ESM.docx]

**Table 1: GRIPP2 Short Form**

| **Section and topic** | **Item** | **Reported on page No** |
| --- | --- | --- |
| 1. Aim | Report the aim of PPI in the study | 4-5 |
| 1. Methods | Provide a clear description of the methods used for PPI in the study | 10-11 |
| 1. Study results | Outcomes – Report the results of PPI in the study, including both positive and negative outcomes | 10-11 |
| 1. Discussion and conclusions | Outcomes – Comment on the extent to which PPI influenced the study overall. Describe positive and negative effects. | 10-11, 13 |
| 1. Reflections/critical perspective | Comment critically on the study, reflecting on the things that went well and those that did not, so others can learn from this experience | 10-11, 13 |
