# Supplementary material for: ‘Plugging the gap’: development of a plain language glossary for statistical methodology research
Source: Res Involv Engagem. 2025 Oct 14;11:118. doi: 10.1186/s40900-025-00782-4 (PMC12523162; doi:10.1186/s40900-025-00782-4)
Supplement: Supplementary file 2 — Supplementary Material 2 [file 40900_2025_782_MOESM2_ESM.docx]

List of terms defined in the glossary:

1. Aggregate data
2. Agreement (inter-rater reliability)
3. Bayesian
4. Bootstrapping
5. Borrowing strength
6. Burn-in
7. Calibration (of measurements)
8. Calibration (of prognostic models)
9. Causal inference
10. Censoring
11. Competing risks
12. Component network meta-analysis
13. Composite outcome
14. Continuous outcome
15. Convergence
16. Coverage (in a simulation study)
17. Cross validation
18. Diagnostic model
19. Discrimination (for prognostic models)
20. Deviance
21. Electronic health records
22. Estimand
23. External validation
24. Flexible parametric model
25. Frequentist
26. Generalized linear model
27. Granular data
28. Hazard
29. Hazard ratio
30. Individual patient data
31. Internal validation (for prognostic modelling)
32. Interpolation
33. Jackknife
34. Likelihood
35. Linear regression
36. Logistic regression
37. Markov Chain Monte Carlo (MCMC)
38. Mean difference
39. (Statistical) Model
40. Model fit
41. Multiple imputation
42. Multiplicity
43. Multi-state model
44. Network meta-analysis
45. Parametric
46. Pooled estimate
47. Posterior distribution
48. Precision
49. Prior distribution (vague & informative)
50. Prognostic model
51. Regression
52. Residuals
53. Risk difference
54. Risk ratio
55. Shrinkage
56. Simulation study
57. Standardised mean difference
58. Statistical methodology research
59. Surrogate outcome
60. Survival analysis
61. Time-to-event outcome
62. Type 1 error
63. Type 2 error
64. Validation
